# Neighborhood disinvestment and severe maternal morbidity in the state of California

**DOI:** 10.1016/j.ajogmf.2023.100916

**Published:** 2023-03-10

**Authors:** Mahasin S. Mujahid, Elizabeth Wall-Wieler, Elleni M. Hailu, Rachel L. Berkowitz, Xing Gao, Colleen M. Morris, Barbara Abrams, Audrey Lyndon, Suzan L. Carmichael

**Affiliations:** Division of Epidemiology, School of Public Health, University of California, Berkeley, Berkeley, CA (Dr Mujahid, Mses Hailu, Gao, and Morris, and Dr Abrams); Division of Neonatal and Developmental Medicine, Department of Pediatrics, Stanford University (Drs Wall-Wieler and Carmichael); Department of Community Health Sciences, University of Manitoba, Winnipeg, Manitoba, Canada (Dr Wall-Wieler); Division of Health Policy and Management, School of Public Health, University of California, Berkeley, Berkeley, CA (Dr Berkowitz); Rory Meyers College of Nursing, New York University, New York City, NY (Dr Lyndon); Division of Maternal-Fetal Medicine, Department of Obstetrics and Gynecology, Stanford University, Stanford, CA (Dr Carmichael).

**Keywords:** Health equity, Neighborhood health effects, Neighborhood deprivation index, Racial and ethnic disparities, Social determinants of health

## Abstract

**BACKGROUND:**

Social determinants of health, including neighborhood context, may be a key driver of severe maternal morbidity and its related racial and ethnic inequities; however, investigations remain limited.

**OBJECTIVE:**

This study aimed to examine the associations between neighborhood socioeconomic characteristics and severe maternal morbidity, as well as whether the associations between neighborhood socioeconomic characteristics and severe maternal morbidity were modified by race and ethnicity.

**STUDY DESIGN:**

This study leveraged a California statewide data resource on all hospital births at ≥20 weeks of gestation (1997–2018). Severe maternal morbidity was defined as having at least 1 of 21 diagnoses and procedures (eg, blood transfusion or hysterectomy) as outlined by the Centers for Disease Control and Prevention. Neighborhoods were defined as residential census tracts (n=8022; an average of 1295 births per neighborhood), and the neighborhood deprivation index was a summary measure of 8 census indicators (eg, percentage of poverty, unemployment, and public assistance). Mixed-effects logistic regression models (individuals nested within neighborhoods) were used to compare odds of severe maternal morbidity across quartiles (quartile 1 [the least deprived] to quartile 4 [the most deprived]) of the neighborhood deprivation index before and after adjustments for maternal sociodemographic and pregnancy-related factors and comorbidities. Moreover, cross-product terms were created to determine whether associations were modified by race and ethnicity.

**RESULTS:**

Of 10,384,976 births, the prevalence of severe maternal morbidity was 1.2% (N=120,487). In fully adjusted mixed-effects models, the odds of severe maternal morbidity increased with increasing neighborhood deprivation index (odds ratios: quartile 1, reference; quartile 4, 1.23 [95% confidence interval, 1.20–1.26]; quartile 3, 1.13 [95% confidence interval, 1.10–1.16]; quartile 2, 1.06 [95% confidence interval, 1.03–1.08]). The associations were modified by race and ethnicity such that associations (quartile 4 vs quartile 1) were the strongest among individuals in the “other” racial and ethnic category (1.39; 95% confidence interval, 1.03–1.86) and the weakest among Black individuals (1.07; 95% confidence interval, 0.98–1.16).

**CONCLUSION:**

Study findings suggest that neighborhood deprivation contributes to an increased risk of severe maternal morbidity. Future research should examine which aspects of neighborhood environments matter most across racial and ethnic groups.

## Introduction

Severe maternal morbidity(SMM), a myriad of unexpected and life-threatening complications related to pregnancy and childbirth, has emerged as a major public health concern. SMM affects 1% to 2% of pregnant individuals (approximately 60,000 people) each year in the United States. Moreover, it has been on the rise for the past 2 decades.^1,2^ There are significant racial and ethnic inequities in SMM, with Black and American Indian or Alaska Native individuals experiencing 2- to 3- fold higher rates of SMM than White individuals.^3–5^ Although extensive research has identified a range of individual, clinical, and hospital factors associated with this increased risk, these factors alone are insufficient in explaining persistent racial and ethnic inequities in SMM.^3,4,6,7^

Recently, there has been a call to action to investigate the multilevel social determinants of SMM.^8–11^ A systematic review identified 83 studies from 1999 to 2018 that examined social factors related to maternal morbidity and mortality and found that 94% of studies focused on individual-level social factors (eg, maternal education and insurance status).^12^ However, neighborhood environments, including their socioeconomic conditions, physical characteristics, and social contexts, may also be important drivers of SMM and SMM inequities. We know that there is substantial geographic variation in SMM and that this variation is more than just a function of the racial and ethnic composition of the region.^13,14^ Alternatively, features of neighborhood environments may affect SMM through several pathways. First, neighborhood environments may influence the risk of preexisting conditions. Studies have shown that individuals residing in adverse neighborhood environments have higher obesity, diabetes mellitus, and other cardio-metabolic risk factors associated with SMM.^15–17^ Second, neighborhood environments may be associated with multiple aspects of pregnancy health, including maternal diet, physical activity, and gestational weight gain.^18–21^ Third, neighborhood environments may affect direct physiological processes tied to chronic stress and accelerated aging.^22,23^ Finally, neighborhood environments may be related to access to and quality of healthcare.^24–26^

Investigations of neighborhood factors concerning SMM are in their infancy. The few existing studies have examined place-based socioeconomic status measures at various scales: county, ZIP code, and New York City community district.^3,27–29^ To date, findings have mostly been null, with only 1 study documenting an association between ZIP code–level median household income and SMM, independent of individual-level confounders.^3^ A major limitation of these studies is the use of large geographic boundaries that introduce a great deal of within-area heterogeneity and mask important geographic differences in risk.^30^ Thus, more research is needed to examine these associations using more granular units, such as census tracts or block groups.

To begin to address these gaps in the literature, we examined associations between neighborhood socioeconomic characteristics, measured at the census tract level, and SMM in the state of California. Given rates of SMM are highest in racially and ethnically marginalized populations, understanding whether adverse neighborhood environments differentially affect SMM in these groups may inform efforts to address disparities in these groups. Thus, we also examined whether associations are modified by race and ethnicity. We hypothesized that birthing people residing in more socioeconomically disadvantaged neighborhoods would have a higher risk of SMM and that these associations would vary substantially by race and ethnicity, with associations being more pronounced among racially and ethnically marginalized individuals.

## Materials and Methods

### Study population

**FIGURE 1:**
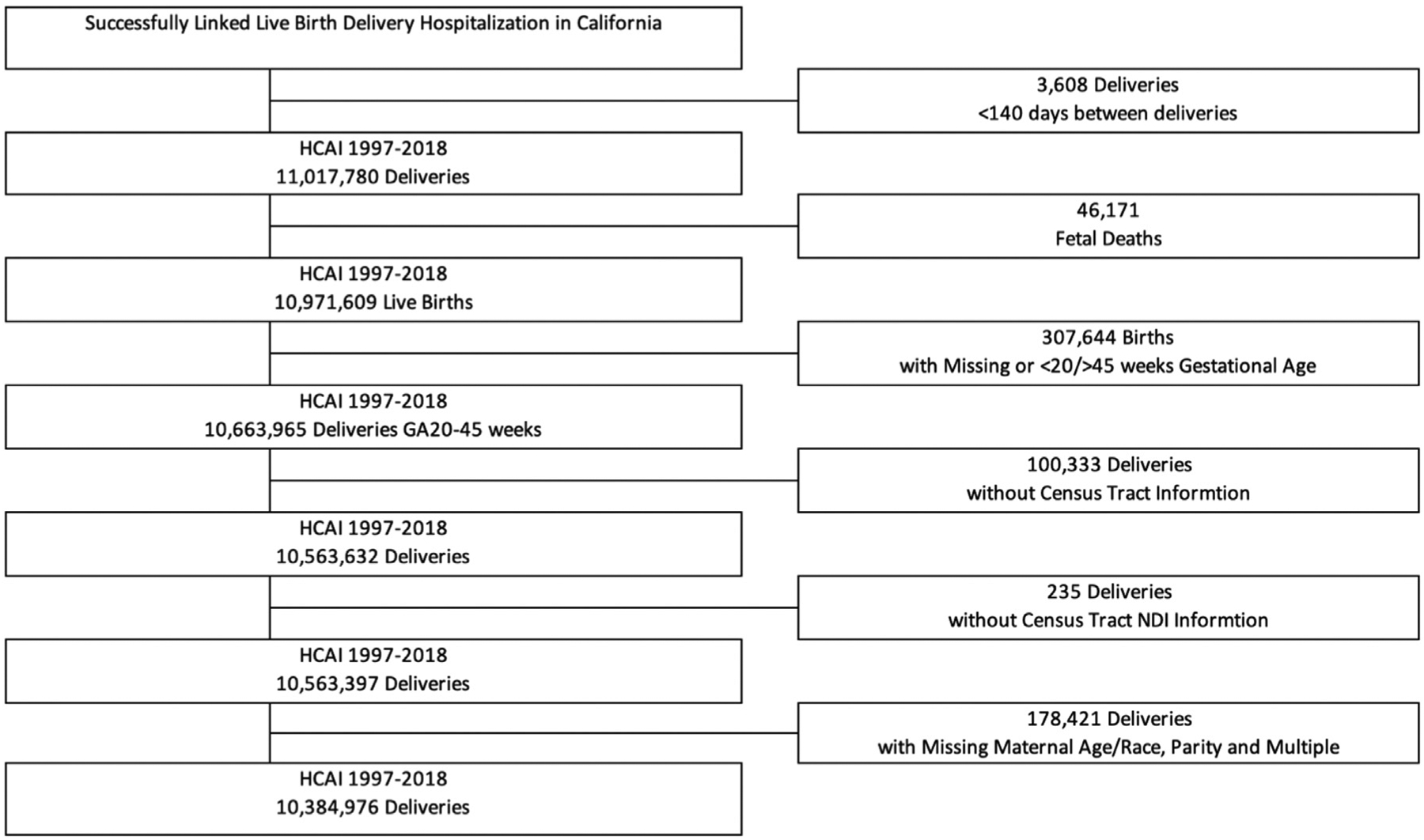
Analytical sample selection, California, 1997–2018 (N=10,384,976) The figure shows the summary of the study exclusion criteria leading to the final analytical sample. After removing samples because of missing or invalid data, the final analytical sample consisted of 10,384,976 births.

Data for this study are from all hospital live births in California from 1997 to 2018, obtained from the California Department of Health Care Access and Information, formerly the Office of Statewide Health Planning and Development, which has linked hospital discharge records with birth certificates (N=10,971,609). We excluded births from our analyses based on the following criteria: missing gestational age or gestational age at <20 or >45 weeks of gestation (n=307,644), data unable to be linked to a census tract (n=100,568), and missing maternal race and ethnicity, parity, and non–first birth for non-singleton delivery (n=178,421). The final analytical sample consisted of 10,384,976 births (Figure 1). The study protocol study was approved by the state of California Committee for the Protection of Human Subjects and the institutional review boards of Stanford University and the University of California, Berkeley.

### Study outcome

We assessed SMM during birth hospitalization using the US Centers for Disease Control and Prevention SMM Index, which is validated for use with administrative and population surveillance data.^2,31^ The SMM index contains 21 indicators related to life-threatening diagnoses and procedures (eg, heart failure, temporary tracheostomy, and transfusion). These indicators were obtained from hospital discharge records using the International Classification of Disease, Ninth Revision, Clinical Modification and the International Classification of Disease, Tenth Revision, Clinical Modification and diagnostic and procedure codes (Supplemental Table 1). Individuals whose hospital discharge records contained one or more of these 21 indicators were categorized as having SMM.

In sensitivity analyses, we excluded individuals with blood transfusion as their only SMM indicator because they might not all represent true cases of SMM, given that information on the number of units of blood transfusion was not available.^31,32^

### Standardized neighborhood deprivation index

Neighborhoods were defined as census tracts. Based on previous work, we constructed a neighborhood deprivation index (NDI) to investigate the association between neighborhood socioeconomic context and SMM. We consider NDI to be a proxy for a broad range of specific features of neighborhood environments, which may provide general insights into the potential effects of neighborhood environments on SMM. The index, originally developed by Messer et al^33^ and widely used in the maternal and infant health literature,^34^ combined 8 census tract variables: percentage of adults in management and professional occupations, percentage of crowded households, percentage of households in poverty, percentage of female-headed households with dependents, percentage of households on public assistance, percentage of households earning <$30,000, percentage of adults with less than a high school diploma, and percentage of adults unemployed.^33^ This index was standardized with a mean of 0 and a standard deviation (SD) of 1, with higher NDI scores indicating more deprivation and lower NDI scores indicating less deprivation. For births between 1997 and 2004, these variables were extracted from data from the 2000 census. For births between 2005 and 2010, NDI variables were characterized using 2005–2010 American Community Survey (ACS) 5-year estimates. Similarly, births between 2011 and 2015 were linked to 2011–2015 ACS estimates, and births between 2016 and 2017 were linked to 2015–2019 ACS estimates. In our analyses, we categorized this continuous score into quartiles for the 1997–2004 and 2005–2018 births separately (quartile 1 [low deprivation] to quartile 4 [high deprivation]). Across the state of California, there were 8022 neighborhoods, with an average of 1295 births per neighborhood. The census tract 2000 boundary was normalized to 2010 using the Longitudinal Tract Database.^35,36^

### Race and ethnicity

Maternal race or ethnicity was determined from birth certificates and categorized as Non-Hispanic White (hereafter, White), Non-Hispanic Black (hereafter, Black), Hispanic, Asian or Pacific Islander (Asian Indian, Chinese, Filipino, Japanese, Korean, Vietnamese, Native Hawaiian, Guamanian or Chamorro, Samoan, other Asian, or other Pacific Islander; hereafter, Asian or Pacific Islander), and other race and ethnicity. Because of the small sample sizes, we combined birthing people who were identified as American Indian or Alaska Native (n=44,199 [0.4%]) with other and mixed-race group (n=6807 [0.1%]) to create an “other” race and ethnicity category.

### Covariates

Based on previous literature, we examined an extensive list of maternal and clinical factors as confounders, using data from birth certificates and hospital discharge records. Maternal demographic and pregnancy-related characteristics assessed included maternal education (high school education or less, some college, or completed college), primary method used for childbirth payment (Medi-Cal, private insurance, or other or unknown), maternal age at childbirth (<20, 20 to 34, or ≥35 years), plurality (singleton or multiple), and parity (any vs no previous live birth). The clinical comorbidity score (continuous) was estimated from 26 comorbidities with International Classification of Diseases Clinical Modification codes, which were assigned weighted values based on their ability to predict SMM.^37^

### Statistical analyses

All statistical analyses were performed using SAS (version 9.4; SAS Institute Inc, Cary, NC). In descriptive analyses, we compared the distribution of study covariates by NDI and SMM and reported proportions, means, and SDs. To determine whether NDI was associated with SMM, we used a series of mixed-effects logistic regression models with individuals nested within neighborhoods. As SMM is a rare outcome, we reported odds ratios (ORs) as suitable approximations of relative risk.^38^ The unadjusted model included only NDI (model 1), and additional models sequentially included maternal sociodemographic characteristics, pregnancy-related factors, and clinical comorbidities (model 2) and maternal race and ethnicity (model 3). To examine whether associations between NDI and SMM were modified by race and ethnicity, we created a cross-product term, and interactions with a *P* value of <.05 were considered statistically significant.

## Results

Among 10,384,976 births, 120,487 (1.2%) were SMM births, and the mean maternal age was 28.4 (SD, 6.26). The distribution of maternal race and ethnicity was 50.8% Hispanic, 29.5% White, 13.5% Asian or Pacific Islander, 5.8% Black, and 0.5% individuals in the “other” racial and ethnic category. Table 1 shows the distribution of maternal characteristics overall and by quartiles of neighborhood disadvantage. Compared with the overall population, individuals living in the highest quartile of NDI (ie, most deprived) were more likely to identify as Black (8.1% [the highest quartile] vs 5.8% [overall]) and Hispanic (75.7% [the highest quartile] vs 50.8% [overall]) and less likely to be White (9.8% [highest quartile] vs 29.5% [overall]). Moreover, compared with the overall population, individuals living in the highest quartile of NDI had a higher representation of individuals with less than a high school education (74.2% [the highest quartile] vs 50.9% [overall]) and individuals with Medi-Cal (72.1% [the highest quartile] vs 46.5% [overall]).

The incidence of SMM increased with increasing neighborhood disadvantage: 102.9 per 10,000 in quartile 1, 109.3 per 10,000 in quartile 2, 115.4 per 10,000 in quartile 3, and 127.1 per 10,000 in quartile 4 (Table 2). In mixed-effects unadjusted models, individuals living in neighborhoods with more disadvantage (ie, quartile 2–quartile 4 compared with quartile 1) had 5% to 23% higher odds of SMM, conditional on the random effect for neighborhood (all confidence intervals [CIs] excluded the null). The associations persisted after adjustment for maternal age, education, insurance type, parity, plurality, and clinical comorbidities (model 2) (Table 2) and became slightly attenuated after adjustment for maternal race and ethnicity (model 3: quartile 2, 1.04 [95% CI, 1.01–1.06]; quartile 3, 1.07 [95% CI, 1.05–1.10]; quartile 4, 1.14 [95% CI, 1.11–1.17]) (Table 2).

**FIGURE 2:**
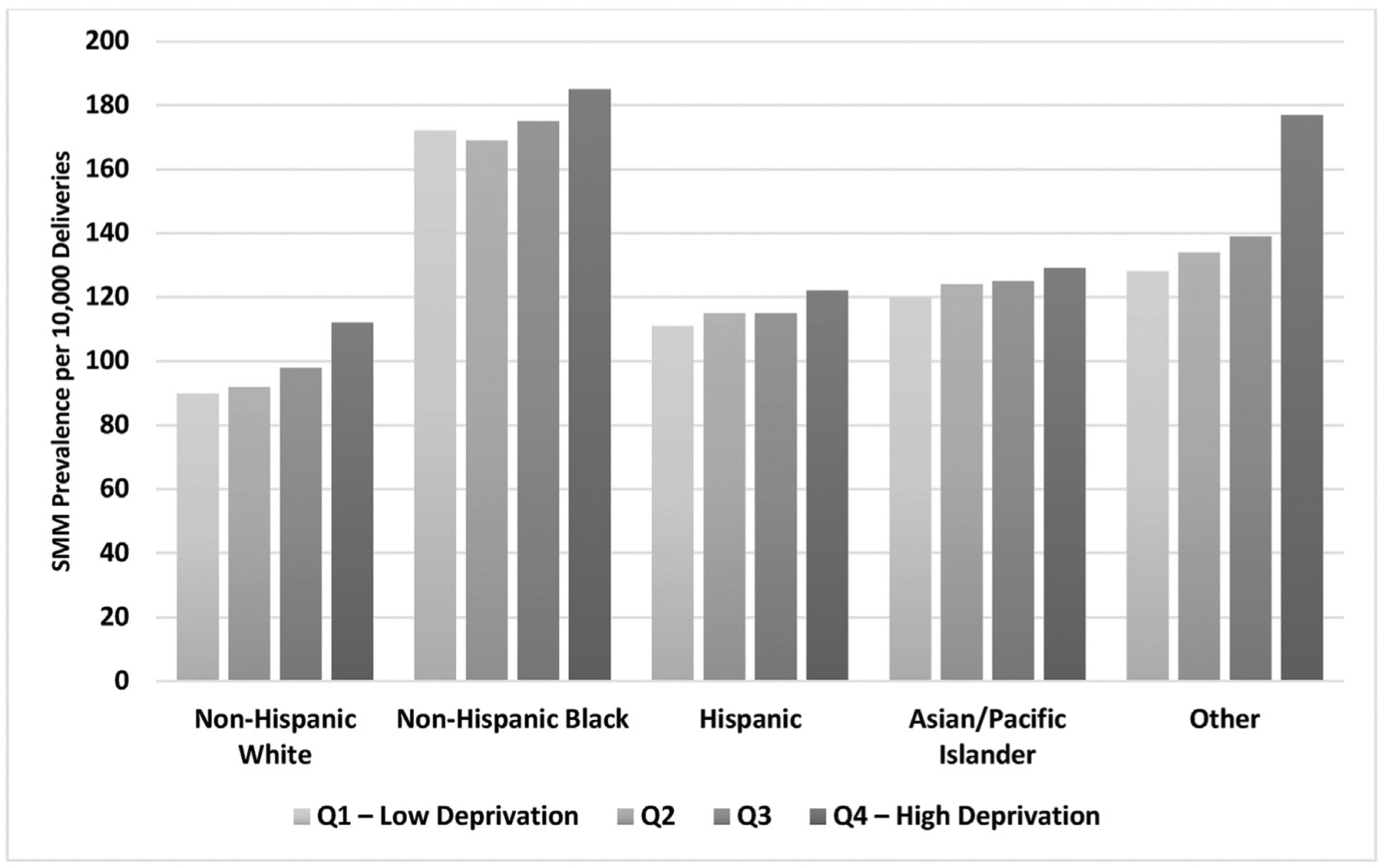
SMM prevalence across neighborhood deprivation by race and ethnicity, California, 1997–2018 (n=10,384,976) The figure shows the SMM prevalence per 10,000 deliveries across neighborhood deprivation by race and ethnicity. “Other” refers to individuals who were identified as American Indian or Alaska Native, mixed race, and other. Black individuals had the highest prevalence of SMM across all quartiles. *SMM*, severe maternal morbidity.

A further examination of the interplay between neighborhood disadvantage and race and ethnicity revealed a significant interaction in unadjusted (*P*<.0001) (Figure 2) and fully adjusted models (*P*<.001) (Table 3). The association between NDI and SMM among White (quartile 2, 1.04 [95% CI, 1.01–1.07]; quartile 3, 1.10 [95% CI, 1.07–1.14]; quartile 4, 1.22 [95% CI, 1.17–1.27]), Hispanic (quartile 2, 1.08 [95% CI, 1.03–1.13]; quartile 3, 1.10 [95% CI, 1.06–1.14]; quartile 4, 1.17 [95% CI, 1.12–1.22]), and Asian or Pacific Islander (quartile 2, 1.05 [95% CI, 1.01–1.09]; quartile 3, 1.07 [95% CI, 1.02–1.12]; quartile 4, 1.14 [95% CI, 1.08–1.20]) individuals was similar in direction and magnitude, with a clear gradient of increasing SMM with increasing NDI as seen in the total population. Among those in the “other” racial and ethnic category, individuals living in the highest quartile of NDI (1.39; 95% CI, 1.39–1.86) had a higher risk of SMM than those living in the lowest quartile of NDI. However, there was no statistically significant association between NDI and SMM among Black individuals

In sensitivity analyses, the associations between NDI and SMM were comparable for nontransfusion SMM in the overall models, although results had less precision as demonstrated by the wider CIs (Supplemental Table 2). Race- and ethnicity-stratified models showed that the associations between some levels of NDI and nontransfusion SMM were attenuated for some groups, specifically Hispanic, Asian, and other racial groups.

## Comment

### Principal findings

Our analysis of the relationship between neighborhood disadvantage and SMM in a statewide sample of 10.4 million births in California from 1997 to 2018 found that SMM risk was the highest among birthing people who lived in the most deprived areas and that the odds of SMM increased as neighborhood deprivation increased, independent of maternal sociodemographic characteristics, pregnancy-related factors, and comorbidities. This pattern was observed for all racial and ethnic groups, with the strongest association among individuals in the “other” racial and ethnic category; however, associations were not statistically significant for Black individuals.

### Results

Our results provided evidence that neighborhood deprivation, measured at the census tract level, influences SMM. Other studies have characterized contextual socioeconomic status at broader geographic scales, including county-level socioeconomic status indicators, ZIP code–level household income quartile, and community district–level poverty.^13,27–29^ County-level socioeconomic characteristics were not associated with SMM in a study of New York State births,^3,27^ whereas ZIP code–level income, which defined neighborhoods at a comparatively finer scale than county-level income, was inversely associated with rates of SMM in a multistate study.^3,27^ Community district–level poverty in New York City, a scale that fell between county and ZIP code, was not statistically significantly associated with SMM overall but modified SMM risk such that residence in a high-poverty neighborhood significantly increased the SMM risk difference between Black and White birthing people and between Hispanic and White birthing people, compared with this difference in wealthier districts.^28^ Finally, we found that the associations between NDI and SMM persisted after adjusting for individual-level socioeconomic factors and pregnancy-related clinical factors, suggesting that other mechanisms, such as quality of care during delivery or exposure to discrimination, may be operating to influence SMM risks.

In addition, we found that the association between neighborhood deprivation and SMM was modified by race and ethnicity. Among White, Hispanic, and Asian or Pacific Islander individuals and those in the “other” racial and ethnic category, living in neighborhoods with a higher NDI was associated with higher odds of SMM. It is rare to have a sufficient sample size in neighborhood health effects research to explore these cross-level interactions, and our findings that associations are strong among Hispanic, Asian or Pacific Islander, and especially “other” (predominately Native American) individuals contribute to a limited literature on multilevel predictors of reproductive outcomes among these groups.

However, we also found that there was no statistically significant association between NDI and SMM among Black individuals. Although this was counter to our a priori hypotheses, it is not unprecedented. Evidence regarding the relationship between neighborhood characteristics and reproductive outcomes among Black individuals is mixed.^39,40^ Of note, 1 study that examined preterm birth (PTB) outcomes found that White and Black individuals in the same geographic areas had a different relationship between NDI and PTB; although living in a more deprived neighborhood significantly increased the risk of PTB for White individuals in 7 of 8 geographies, significantly increased risk of PTB was only seen for Black individuals in 2 of 8 geographies and at a lower magnitude than observed for White individuals.^41^ In another study, significant relationships were found between specific characteristics of neighborhood deprivation (eg, physical incivilities or walkability) and low birthweight and PTB for White but not Black individuals.^42^ It is possible that for Black individuals, factors not measured in this study but known to impact chronic stress and reproductive outcomes (eg, experiences of racism or social support) modify the relationship between NDI and SMM.^34,43–46^ For example, Black individuals living in high NDI neighborhoods with high concentrations of chronic stressors may benefit from strong social support networks that mitigate the negative effects of living in a deprived neighborhood.^47^ Black individuals living in lower NDI neighborhoods may be more likely to experience racial discrimination and social exclusion, nullifying the protective effect that a less deprived neighborhood may provide for other racial groups.^48^ Future research is needed to understand the pathways through which neighborhoods may differentially affect the risk of SMM across different groups of Black individuals.

### Clinical implications

Our findings underscored the need to move beyond pregnancy-related factors and clinical comorbidities and considered a broader range of social determinants of health (eg, neighborhood context) to fully understand and address the etiology of SMM and SMM disparities. The collection and inclusion of information on patients’ neighborhood context in electronic health records and hospital discharge data would be a crucial step in this direction.

### Research implications

As our findings suggested that the scale at which neighborhood context is measured may matter, future research should examine associations at more granular levels to uncover geographic differences in risk that may be masked at the county or ZIP code level. To facilitate this research aim, clinical databases should leverage patient address data to allow for geocoding at the neighborhood level.

As there are persistent racial and ethnic inequities in SMM, future research is needed to expand the measurement of both harmful and protective neighborhood-level factors that may be more salient for racially and ethnically marginalized populations and to examine their effect on SMM.

### Strengths and limitations

This study examined the relationship between NDI and SMM using statewide data in California and a more granular geographic definition of neighborhood at the census tract level, a more comprehensive characterization of neighborhood disadvantage, and a larger sample size than most previous studies. The large population of Asian or Pacific Islander and Hispanic individuals in our dataset provided an important opportunity for us to examine associations by race and ethnicity that may not be possible using other datasets with smaller sample sizes of these groups.

Several limitations warranted comment. First, although we used a validated measure of SMM, there may be a misclassification of our outcome. Potential sources of misclassification included the underreporting of rare SMM conditions (eg, eclampsia and other cardiac and renal conditions) in hospital discharge data and the classification of SMM for individuals who received a blood transfusion for nonsevere complications.^31,49^ In sensitivity analyses, we found comparable associations between neighborhood disadvantage and SMM, mitigating concern regarding the latter source of bias. Second, our use of census tracts as proxies for neighborhoods may be limiting; although census tracts are designed to be more socioeconomically homogenous than larger census-defined boundaries, they may not reflect boundaries that are meaningful to residents or the underlying boundaries of spatial inequities. A related concern is the absence of direct measurement of specific features of neighborhood environments and the use of neighborhood deprivation as a crude proxy for features, such as high crime and other place-based stressors that may matter for SMM. Future studies should examine specific features that may be more amenable to change. Finally, although we controlled for an extensive list of covariates, we cannot rule out the possibility of residual confounding because of the unavailability of data on individual-level measures (eg, household income).

### Conclusions

Leveraging one of the largest statewide databases, we found that neighborhood disadvantage was associated with SMM at the census tract level. Furthermore, NDI influenced SMM risk among White, Asian or Pacific Islander, Hispanic, and other racial groups. This work provides support for the crucial impact of social determinants of health, operating at multiple levels, on maternal health inequities.

## Supplementary Material

supplementary material

## Figures and Tables

**TABLE 1:** Distribution of maternal characteristics by neighborhood deprivation index quartiles, California, 1997–2018 (N=10,384,976)

Maternal characteristics	All, n (%)	Neighborhood deprivation quartiles,^a^ n (%)			
		Quartile 1 (n=1,817,201)	Quartile 2 (n=2,195,976)	Quartile 3 (n=2,736,997)	Quartile 4 (n=3,634,802)
Race and ethnicity					
Non-Hispanic White	3058,811 (29.5)	1,023,796 (56.3)	960,066 (43.7)	719,740 (26.3)	355,209 (9.8)
Non-Hispanic Black	601,503 (5.8)	45,038 (2.5)	96,443 (4.4)	165,455 (6.0)	294,567 (8.1)
Hispanic	5,274,129 (50.8)	296,099 (16.3)	728,772 (33.2)	1,497,326 (54.7)	2,751,932 (75.7)
Asian or Pacific Islander	1,399,527 (13.5)	446,329 (24.6)	398,834 (18.2)	336,792 (12.3)	217,572 (6.0)
Other	51,006 (0.5)	5939 (0.3)	11,861 (0.5)	17,684 (0.6)	15,522 (0.4)
Age (y)					
<20	877,768 (8.5)	35,534 (2.0)	113,457 (5.2)	251,075 (9.2)	477,702 (13.1)
20–34	7,638,780 (73.6)	1,199,995 (66.0)	1,629,178 (74.2)	2,082,402 (76.1)	2,727,205 (75.0)
≥35	1,868,428 (18.0)	581,672 (32.0)	453,341 (20.6)	403,520 (14.7)	429,895 (11.8)
Education					
High school education or less	5,289,687 (50.9)	291,959 (16.1)	770,600 (35.1)	1,530,647 (55.9)	2,696,481 (74.2)
Some college	2,318,074 (22.3)	365,626 (20.1)	599,651 (27.3)	698,359 (25.5)	654,438 (18.0)
College graduate	2,595,070 (25.0)	1,128,155 (62.1)	787,513 (35.9)	461,471 (16.9)	217,931 (6.0)
Missing or unknown	182,145 (1.8)	31,461 (1.7)	38,212 (1.7)	46,520 (1.7)	65,952 (1.8)
Payment type at delivery					
Medi-Cal	4,824,537 (46.5)	207,100 (11.4)	631,184 (28.7)	1,365,499 (49.9)	2,620,754 (72.1)
Private	5,207,585 (50.1)	1,542,133 (84.9)	1,482,322 (67.5)	1,274,250 (46.6)	908,880 (25.0)
Other or unknown	352,854 (3.4)	67,968 (3.7)	82,470 (3.8)	97,248 (3.6)	105,168 (2.9)
Multiple pregnancy					
Yes	156,640 (1.5)	40,929 (2.3)	37,007 (1.7)	36,623 (1.3)	42,081 (1.2)
No	10,228,336 (98.5)	1,776,272 (97.7)	2,158,969 (98.3)	2,700,374 (98.7)	3,592,721 (98.8)
Primiparous					
Yes	4,018,861 (38.7)	814,028 (44.8)	940,253 (42.8)	1,053,505 (38.5)	1,211,075 (33.3)
No	6,366,115 (61.3)	1,003,173 (55.2)	1,255,723 (57.2)	1,683,492 (61.5)	2,423,727 (66.7)
Comorbidities^b^	8.0 (14.7)	7.9 (15.1)	8.1 (14.9)	7.9 (14.6)	7.9 (14.4)

Clinical comorbidities score is equal to weighted average of 26 comorbidities.

a Quartile 1 represents the lowest deprivation neighborhoods, and quartile 4 represents the highest (worst) deprivation neighborhoods;

b Data are presented as mean (standard deviation) for the continuous comorbidity score.

**TABLE 2:** SMM prevalence, unadjusted OR, and adjusted OR of SMM by neighborhood deprivation index quartile, California, 1997–2018 (N=10,384,976)

Neighborhood deprivation	Deliveries	SMM cases	SMM prevalence per 10,000 deliveries	Unadjusted OR (95% CI), without neighborhood mixed effect	Including neighborhood mixed effect		
					Unadjusted OR (95% CI)	Adjusted OR (95% CI)^a^	Adjusted OR (95% CI)^b^
Q1 (low deprivation)	1,817,201	18,690	102.9	Reference	Reference	Reference	Reference
Q2	2,195,976	23,997	109.3	1.06 (1.04–1.08)	1.05 (1.04–1.08)	1.06 (1.03–1.08)	1.04 (1.01–1.06)
Q3	2,736,997	31,593	115.4	1.12 (1.10–1.14)	1.12 (1.10–1.14)	1.13 (1.10–1.16)	1.07 (1.05–1.10)
Q4 (high deprivation)	3,634,802	46,207	127.1	1.24 (1.22–1.26)	1.23 (1.20–1.26)	1.23 (1.20–1.26)	1.14 (1.11–1.17)

*CI*, confidence interval; *OR*, odds ratio; *Q*, quartile; *QSMM*, severe maternal morbidity.

a The data have been adjusted for maternal age, education, insurance type, parity, plurality, and comorbidity score;

b The data have been adjusted for maternal age, education, insurance type, parity, plurality, comorbidity score, and race and ethnicity.

**TABLE 3:** Prevalence, unadjusted OR, and adjusted OR of SMM by NDI quartile and maternal race and ethnicity, California, 1997–2018 (n=10,384,976)

NDI quartile and maternal race and ethnicity	Deliveries	SMM cases	SMM prevalence per 10,000 deliveries	Unadjusted OR (95% CI)	Adjusted OR (95% CI)^a^
Model 1: non-Hispanic White (n=2,300,151)					
Q1 (low deprivation)	1,023,796	9198	90	Reference	Reference
Q2	960,066	8844	92	1.01 (0.97–1.05)	1.04 (1.01–1.07)
Q3	719,740	7024	98	1.03 (0.99–1.08)	1.10 (1.07–1.14)
Q4 (high deprivation)	355,209	3964	112	1.12 (1.06–1.18)	1.22 (1.17–1.27)
Model 2: non-Hispanic Black (n=453,856)					
Q1 (low deprivation)	45,038	776	172	Reference	Reference
Q2	96,443	1634	169	0.96 (0.85–1.08)	1.00 (0.92–1.10)
Q3	165,455	2903	175	0.96 (0.86–1.07)	1.03 (0.94–1.12)
Q4 (high deprivation)	294,567	5457	185	1.01 (0.90–1.12)	1.07 (0.98–1.16)
Model 3: Hispanic (n=3,970,402)					
Q1 (low deprivation)	296,099	3300	111	Reference	Reference
Q2	728,772	8404	115	0.96 (0.91–1.02)	1.08 (1.03–1.13)
Q3	1,497,326	17,217	115	0.94 (0.89–1.00)	1.10 (1.06–1.14)
Q4 (high deprivation)	2,751,932	33,704	122	0.97 (0.93–1.03)	1.17 (1.12–1.22)
Model 4: Asian or Pacific Islander (n=967,753)					
Q1 (low deprivation)	446,329	5340	120	Reference	Reference
Q2	398,834	4956	124	1.04 (0.98–1.10)	1.05 (1.01–1.09)
Q3	336,792	4203	125	1.00 (0.94–1.06)	1.07 (1.02–1.12)
Q4 (high deprivation)	217,572	2807	129	1.00 (0.93–1.07)	1.14 (1.08–1.20)
Model 5: other (n=37,614)					
Q1 (low deprivation)	5939	76	128	Reference	Reference
Q2	11,861	159	134	1.03 (0.70–1.52)	1.07 (0.79–1.44)
Q3	17,684	246	139	0.82 (0.56–1.20)	1.12 (0.83–1.50)
Q4 (high deprivation)	15,522	275	177	1.09 (0.75–1.58)	1.39 (1.03–1.86)

*CI*, confidence interval; *NDI*, neighborhood deprivation index; *OR*, odds ratio; *Q*, quartile; *SMM*, severe maternal morbidity.

a Data have been adjusted for maternal age, education, insurance type, parity, plurality, and comorbidity score.

## Why was this study conducted?

Neighborhood environments may be important drivers of severe maternal morbidity (SMM); however, research is needed to investigate these associations at a more granular census tract level and to examine whether associations are modified by race and ethnicity, given that rates of SMM are the highest in racially and ethnically marginalized populations.

## Key findings

The odds of SMM increased with increasing neighborhood deprivation, independent of maternal sociodemographic and pregnancy-related factors and comorbidities.

## What does this add to what is known?

This work contributes evidence that neighborhood deprivation influences SMM and suggests that census tract level may be a meaningful scale to assess how contextual disadvantages influence pregnancy-related outcomes.
