# Supplementary material for: Neighborhood disinvestment and severe maternal morbidity in the state of California
Source: Am J Obstet Gynecol MFM. Author manuscript; Available in PMC 2024 Mar 22. (PMC10959123; doi:10.1016/j.ajogmf.2023.100916)
Supplement: supplementary material [file NIHMS1973419-supplement-supplementary_material.docx]

**Supplementary Table 1: SMM Indicators and ICD-9 Codes**

| Severe Maternal Morbidity Indicator | DX or PR | ICD-9 | ICD-10 |
| --- | --- | --- | --- |
| 1. Acute myocardial infraction | DX | 410.xx | I21.xx, I22.x |
| 2. Aneurysm | DX | 441.xx | I71.xx*, I79.0 |
| 3. Acute renal failure | DX | 584.5, 584.6, 584.7, 584.8, 584.9, 669.3x | N17.x, O90.4 |
| 4. Adult respiratory distress syndrome | DX | 518.5x, 518.81 518.82 518.84, 799.1 | J80, J95.1, J95.2, J95.3, J95.82x, J96.0x, J96.2x R09.2 |
| 5. Amniotic fluid embolism | DX | 673.1x | O88.1x |
| 6. Cardiac arrest/ventricular fibrillation | DX | 427.41, 427.42*, 427.5 | I46.x, I49.0x |
| 7. Conversion of cardiac rhythm | PR | 99.6x | 5A2204Z, 5A12012 |
| 8. Disseminated intravascular coagulation | DX | 286.6, 286.9, 666.3x | D65, D68.8, D68.9, O72.3 |
| 9. Eclampsia | DX | 642.6x | O15. X |
| 10. Heart failure/arrest during surgery or procedure | DX | 997.1 | I97.12x, I97.13x, I97.710, I97.711 |
| 11. Puerperal cerebrovascular disorders | DX | 430.xx, 431.xx, 432.xx, 433.xx, 434.xx, 436xx, 437.xx, 671.5x, 674.0x, 997.02 | I60.xx- I68.xx, O22.51, O22.52, O22.53, I97.81x, I97.82x, O87.3 |
| 12. Pulmonary edema / Acute heart failure | DX | 518.4, 428.1, 428.0, 428.21, 428.23, 428.31, 428.33, 428.41, 428.43 | J81.0, I50.1, I50.20, I50.21, I50.23, I50.30, I50.31, I50.33, I50.40, I50.41, I50.43, I50.9 |
| 13. Severe anesthesia complications | DX | 668.0x*, 668.1x, 668.2x | O74.0 , O74.1 , O74.2, O74.3, O89.0x, O89.1, O89.2 |
| 14. Sepsis | DX | 038.xx, 995.91, 995.92, 670.2x (after October 1, 2009) | O85, O86.04, T80.211A, T81.4XXA, T81.44xx, **OR** R65.20 **OR** A40.x, A41.x, A32.7 |
| 15. Shock | DX | 669.1x, 785.5x, 995.0, 995.4, 998.0x | O75.1, R57.x, R65.21, T78.2XXA, T88.2 XXA, T88.6 XXA, T81.10XA , T81.11XA, T81.19XA |
| 16. Sickle cell disease with crisis | DX | 282.42, 282.62, 282.64, 282.69 | D57.0x, D57.21x, D57.41x, D57.81x |
| 17. Air and thrombotic embolism | DX | 415.1x, 673.0x, 673.2x, 673.3x, 673.8x | I26.x, O88.0x, O88.2x, O88.3x, O88.8x |
| 18. Blood products transfusion | PR | 99.0x | 30233H1, 30233L1, 30233K1, 30233M1, 30233N1, 30233P1, 30233R1, 30233T1, 30233H0, 30233L0, 30233K0, 30233M0, 30233N0, 30233P0, 30233R0, 30233T0, 30230H1, 30230L1, 30230K1, 30230M1, 30230N1, 30230P1, 30230R1, 30230T1, 30230H0, 30230L0, 30230K0, 30230M0, 30230N0, 30230P0, 30230R0, 30230T0, 30240H1, 30240L1, 30240K1, 30240M1, 30240N1, 30240P1, 30240R1, 30240T1, 30240H0, 30240L0, 30240K0, 30240M0, 30240N0, 30240P0, 30240R0, 30240T0, 30243H1, 30243L1, 30243K1, 30243M1, 30243N1, 30243P1, 30243R1, 30243T1, 30243H0, 30243L0, 30243K0, 30243M0, 30243N0, 30243P0, 30243R0, 30243T0, 30250H1, 30250L1, 30250K1, 30250M1, 30250N1, 30250P1, 30250R1, 30250T1, 30250H0, 30250L0, 30250K0, 30250M0, 30250N0, 30250P0, 30250R0, 30250T0, 30253H1, 30253L1, 30253K1, 30253M1, 30253N1, 30253P1, 30253R1, 30253T1, 30253H0, 30253L0, 30253K0, 30253M0, 30253N0, 30253P0, 30253R0, 30253T0, 30260H1, 30260L1, 30260K1, 30260M1, 30260N1, 30260P1, 30260R1, 30260T1, 30260H0, 30260L0, 30260K0, 30260M0, 30260N0, 30260P0, 30260R0, 30260T0, 30263H1, 30263L1, 30263K1, 30263M1, 30263N1, 30263P1, 30263R1, 30263T1, 30263H0, 30263L0, 30263K0, 30263M0, 30263N0, 30263P0, 30263R0, 30263T0 |
| 19. Hysterectomy | PR | 68.3x-68.9x | 0UT90ZZ, 0UT94ZZ, 0UT97ZZ, 0UT98ZZ, 0UT9FZZ, 0UT90ZL |
| 20. Temporary tracheostomy | PR | 31.1 | 0B110Z, 0B110F, 0B113, 0B114 |
| 21. Ventilation | PR | 93.90, 96.01, 96.02, 96.03, 96.05 | 5A1935Z, 5A1945Z, 5A1955Z |

DX=diagnostic code

PR=procedure code

**Supplemental Table 2.** SMM Prevalence, Unadjusted and Adjusted Odds Ratios of SMM by Neighborhood Deprivation Index Quartile; California, 1997-2018 (n = 10,384,976) – Excluding blood transfusion-only cases

| Neighborhood Deprivation | Deliveries | SMM Cases | SMM Prevalence per 10,000 Deliveries | Unadjusted OR (95% CI), without neighborhood mixed effect | Including Neighborhood Mixed Effect | | |
| --- | --- | --- | --- | --- | --- | --- | --- |
|  |  |  |  |  | Unadjusted OR (95% CI) | Adjusted OR^a^ (95% CI) | Adjusted OR^b^ (95% CI) |
| Q1 – Low Deprivation | 1,817,201 | 9,163 | 50.4 | Reference | Reference | Reference | Reference |
| Q2 | 2,195,976 | 11,346 | 51.7 | 1.02 (1.00-1.05) | 1.02 (0.99-1.05) | 1.05 (1.02-1.09) | 1.04 (1.00-1.07) |
| Q3 | 2,736,997 | 14,287 | 52.2 | 1.04 (1.01-1.06) | 1.03 (1.00-1.06) | 1.10 (1.07-1.14) | 1.06 (1.02-1.09) |
| Q4 – High Deprivation | 3,634,802 | 20,065 | 55.2 | 1.10 (1.07-1.12) | 1.10 (1.07-1.13) | 1.19 (1.15-1.23) | 1.11 (1.08-1.15) |

SMM=Severe Maternal Morbidity

^a^ Adjusted for maternal age, education, insurance type, parity, plurality, and comorbidity score

^b^ Adjusted for maternal age, education, insurance type, parity, plurality, comorbidity score, and race/ethnicity

**Supplemental Table 3**. Prevalence and Adjusted Odds Ratios of SMM by Neighborhood Deprivation Index Quartile and Maternal Race/Ethnicity; California, 1997-2018 (n = 10,384,976) – Excluding blood transfusion-only cases.

| NDI Quartile and Maternal Race/Ethnicity | Deliveries | SMM Cases | SMM Prevalence per 10,000 Deliveries | Adjusted OR^a^ (95% CI) |
| --- | --- | --- | --- | --- |
| **Model 1: Non-Hispanic White (n = 2,300,151)** |  |  |  |  |
| Q1 – Low Deprivation | 1,023,796 | 4,653 | 45 | Reference |
| Q2 | 960,066 | 4,402 | 46 | 1.04 (0.99-1.08) |
| Q3 | 719,740 | 3,374 | 47 | 1.08 (1.03-1.13) |
| Q4 – High Deprivation | 355,209 | 1,802 | 51 | 1.14 (1.07-1.21) |
| **Model 2: Non-Hispanic Black (n = 453,856)** |  |  |  |  |
| Q1 – Low Deprivation | 45,038 | 403 | 89 | Reference |
| Q2 | 96,443 | 829 | 86 | 1.01 (0.89-1.15) |
| Q3 | 165,455 | 1,422 | 86 | 1.01 (0.90-1.14) |
| Q4 – High Deprivation | 294,567 | 2,649 | 90 | 1.08 (0.97-1.22) |
| **Model 3: Hispanic (n = 3,970,402)** |  |  |  |  |
| Q1 – Low Deprivation | 296,099 | 1,577 | 53 | Reference |
| Q2 | 728,772 | 3,730 | 51 | 1.03 (0.97-1.10) |
| Q3 | 1,497,326 | 7,522 | 50 | 1.05 (0.99-1.12) |
| Q4 – High Deprivation | 2,751,932 | 14,292 | 52 | 1.11 (1.04-1.17) |
| **Model 4: Asian/Pacific Islander (n = 967,753)** |  |  |  |  |
| Q1 – Low Deprivation | 446,329 | 2,492 | 56 | Reference |
| Q2 | 398,834 | 2,307 | 58 | 1.06 (1.00-1.12) |
| Q3 | 336,792 | 1,876 | 56 | 1.05 (0.98-1.12) |
| Q4 – High Deprivation | 217,572 | 1,214 | 56 | 1.09 (1.01-1.18) |
| **Model 5: Other (n = 37,614)** |  |  |  |  |
| Q1 – Low Deprivation | 5,939 | 38 | 64 | Reference |
| Q2 | 11,861 | 78 | 66 | 1.15 (0.75-1.75) |
| Q3 | 17,684 | 93 | 53 | 0.94 (0.62-1.45) |
| Q4 – High Deprivation | 15,522 | 108 | 70 | 1.26 (0.82-1.93) |

SMM=Severe Maternal Morbidity

^a^ Adjusted for maternal age, education, insurance type, parity, plurality, and comorbidity score
